# The experience of patients with hematological malignancy in their terminal stage: a phenomenological study from Jordan’s perspective

**DOI:** 10.1186/s12904-024-01373-y

**Published:** 2024-02-09

**Authors:** Mohammad M. Alnaeem, Anas Shehadeh, Abdulqadir J. Nashwan

**Affiliations:** 1 Palliative Care and Pain Management Program, School of Nursing, Al-Zaytoonah University of Jordan, Airport Street, 11733 Amman, Jordan; 2 Community Health Nursing, School of Nursing, Al-Zaytoonah University of Jordan, Airport Street, 11733 Amman, Jordan; 3 Director of Nursing for Education and Practice Development, Nursing Department, Hamad Medical Corporation, Doha, Qatar

**Keywords:** Hematological malignancy, Palliative Care, Hospice, Phenomenological, Spiritual coping

## Abstract

**Background:**

Patients diagnosed with hematological malignancies residing in low-middle-income countries undergo significant physical and psychological stressors. Despite this, only 16% of them receive proper care during the terminal stages. It is therefore crucial to gain insight into the unique experiences of this population.

**Aim:**

To have a better understanding of the needs and experiences of adult patients with advanced hematological malignancy by exploring their perspectives.

**Methods:**

A qualitative interpretive design was employed to collect and analyze data using a phenomenological approach. The study involved in-depth interviews with ten participants aged between 49 and 65 years, utilizing a semi-structured approach.

**Results:**

Two primary themes emerged from the participants’ experiences of reaching the terminal stage of illness: “Pain, Suffering, and Distress” and “Spiritual Coping.” The first theme encompassed physical and emotional pain, suffering, and distress, while the second theme was centered on the participants’ spiritual coping mechanisms. These coping mechanisms included seeking comfort in religious practices, relying on spiritual support from family and friends, and finding solace in their beliefs and faith.

**Conclusion:**

Patients with hematological malignancies in the terminal stages of their disease experience severe pain, considerable physical and psychosocial suffering, and spiritual distress. While they require support to cope with their daily struggles, their experiences often go unnoticed, leading to disappointment and loss of dignity. Patients mainly rely on their spirituality to cope with their situations. Healthcare providers must acknowledge these patients’ needs and provide more holistic and effective care.

**Supplementary Information:**

The online version contains supplementary material available at 10.1186/s12904-024-01373-y.

## Introduction

Hematological malignancies (HMs) are heterogeneous conditions affecting the bone marrow and lymphatic system. HMs are the sixth most common type of cancer worldwide [1]. Globally in 2020, there were 475,000 new cases of leukemia and over 300,000 reported deaths [1, 2], and more than 44,000 of non-Hodgkin lymphoma [3]. While a tremendous progress was made to enhance the care and longevity of people with HMs, many experience fatigue and poor quality of life as their illness advances [4–7]. Physical symptoms, such as pain, are usually considered the most common symptoms among patients with advanced HMs [6, 8, 9]. Pain was found to hamper self-management [10] and is related to increased mortality [11]. Several studies among patients with HMs showed that the prevalence of pain ranged from 37 to 90% [7, 12, 13].

Patients with HM differ from those with solid cancer in disease trajectory and treatment approaches [14]. Patients with HMs experience frequent hospital admissions, rapid deterioration, often receive aggressive end of life (EOL) care, and rapidly reach the terminal stage of illness [15–17]. Furthermore, patients with an HMs have a short life expectancy, and palliative care services are provided for a short time only [18–21]. These patients suffer from incurable diseases, and are therefore more likely to experience psychological and physical distress. One of the possible causes of suffering is the late stage at which many cancer patients are diagnosed [22]. Based on the literature, terminally ill patients are defined as patients who have a life expectancy of less than six months [23–25].

In Jordan, more than 4,600 new adult patients are diagnosed with cancer annually. However, more than 60% of these cancers are diagnosed in the late stage [22, 26]. There were 477 cases of non-Hodgkin lymphoma and 135 cases of leukemia diagnosed in 2022 [27]. Many of them frequently visit the emergency department and are admitted to hospitals [28], in particular, intensive care units (ICU) [29]. This indicated that most cancer patients were in the terminal stages of illness and spent their last days in stressful environments. Despite providing palliative care services for cancer patients for decades, the services in Jordan are still developing and few patients have access to these services [30, 31]. There is only one private health care center specialized in providing palliative care in Jordan (King Hussein Cancer Center). This presents a severe shortage and lack of accessibility of this type of care, particularly for people with low incomes who only have access to public health care centers.

Due to the focus of research in Jordan on solid cancers, there are few studies conducted among adult patient with HMs. Several studies have reported the negative impacts of cancer on physical, psychosocial, and other aspects of the expereinces of Jordanian patients [7, 28, 32–34]. While many of the physical and psychosocial impacts that patients with solid cancer experience are recognized in the global literature, there is still a need to explore the impacts of HMs [35, 36]. Further exploration of the experience of patients with advanced HMs is therefore required worldwide, particularly among patients living in low and middle-income countries where only 16% of patients receive care in the terminal stages [37].

The aim of this study was to explore the experiences of Jordanian adult patients with HMs and provide original insights about their distressing symptoms that persist throughout the terminal stage of illness. By shedding light on these experiences, we hope that this study can help enhance the understanding of the challenges faced by this patient population and provide valuable information to improve their care and well-being.

## Methods

### Design

The study utilized a qualitative phenomenological inductive approach [38] to explore the experience of patients with HMs in the terminal stages. The study aimed to collect in-depth information about the experience of participants. The primary investigator (MA) conducted one-on-one interviews with the participants following their discharge from hospital to their homes. MA has a clinical experience with different cancer patients in addition to his academic background including a master’s and Ph.D. degrees in palliative cancer care.

### Participants

Patients were selected using a purposeful sampling approach for practical reasons. Participants who met the following inclusion criteria were invited: aged at least 18 years; diagnosed with a HM; aware of the diagnosis; and reached the terminal stage of their disease. Patients who were unable to communicate were excluded from the study. The recruitment process continued until thick descriptions of the same themes were repeatedly emerging [38]. After including ten patients, no new themes, ideas, opinions, or patterns emerged. Three patients refused to participate in this study because they were tired and unable to discuss their issues.

### Settings

This study was conducted in two settings: the first was the oncology department of a large public hospital in the capital of Jordan (Amman), to which all cancer patients are referred to from other hospitals. This department has 20 beds for in-patients and 15 beds for chemotherapy and follow-up. This department provides care for all cancer types, and all primary physicians were oncologists. The other setting was the homes of participants.

### Data collection procedure

Data were collected using face-to-face interviews that asked broad semi-structured questions. Approval from the participating hospital was obtained to access the contact information of potential participants. The first researcher approached potential participants in the hospital. Of those who were able to participate in the study, some were interviewed in the hospital (*n* = 3 patients) and the remaining participants preferred to conduct the interview in their homes (*n* = 7 patients). After being discharged from the hospital, potential participants were contacted via phone to determine the appropriate time for the interview. The participants were reminded via a phone call one day before the interview. Patients chose the time of the interview based on their preferences. Those who agreed were interviewed after providing both verbal permission and written consents. The length of the interviews ranged from 60 to 100 min.

During each interview, the interviewer meticulously noted down non-verbal cues and gestures, capturing nuanced details of the conversation to gain deeper insights into each participant’s perspective. These comprehensive field notes contained the interviewer’s observations, enriching the data with vivid descriptions of the atmosphere and interactions [30]. These detailed records played a crucial role in the subsequent analysis phase by allowing the inclusion of thoughts and emerging insights to enhance the depth of understanding. Prior to the interviews, various elements including medication packaging, intravenous fluid stands used for administering medication such as paracetamol, and religious items such as the Quran, Masbaha*, and prayer rugs were carefully noticed in the participants’ homes. These observations aimed to contextualize the interview data provided by the participants. The detailed field notes from these observations were integrated into the subsequent analysis and reporting processes.

The interview questions for this study were formulated carefully by the authors and are provided in Supplementary File 1, which includes several probe questions used to obtain further clarifications. The researcher employed active listening techniques to promote understanding, and silence to give the participants adequate time to answer. At the end of the interviews, the participants were encouraged to talk about any aspect of their experiences that was not discussed, and they were thanked for their participation.

### Data analysis

The interviews were audio-recorded, transcribed verbatim, and analyzed in Arabic (the participants’ mother language). Each researcher in this study read each interview transcription several times to grasp the meaning of the participants’ experiences and to identify emerging categories [39]. Two researchers, MMA and AS, thoroughly acquainted themselves with the entire dataset. After an initial review of the transcripts, they established a repository of codes. Subsequently, both researchers independently applied these codes to the transcripts and cross-compared their analyses, addressing any disparities through collaborative discussions. The first researcher, MMA, structured the coded data into overarching themes, which underwent evaluation by a secondary reviewer, AS. Any differences in perspectives were reconciled through further deliberation on the identified themes. The ATLAS.ti software version 20 [40] was used to facilitate the organization and coding of the data. The preliminary themes underwent refinement following extensive discussions involving all authors.

To mitigate potential biases, the first researcher maintained a research diary throughout the process. The authors utilized inductive coding in which the first-level coding involved line-by-line analysis of the texts, and the data that represented similar concepts were colored and given the same codes. Then, the codes were categorized and clustered based on their similarity. The final categories that described the essence of the participants’ experiences were framed into exclusive themes and subthemes. The researchers met several times to discuss the emerging themes and agreed on the final categorization. Lastly, the themes were translated into English. Moustakas’s (1994) framework for data collection and analysis was utilized to guide the interpretation of the phenomena under investigation [41].

### Ethical considerations

The Institutional Review Board (IRB) at the University of Jordan and the participating hospital approved this study. All potential participants were informed that participation was voluntary and that there were no consequences if they chose to decline participation. Those who agreed to participate were informed about their right to withdraw from participation at any time without any consequences. Also, a full explanation of the possibility of any potential risks and benefits was declared. Participants were interviewed alone to maintain their confidentiality and privacy. The participants were assigned numbers to protect their identities, (e.g., P1: Participant 1). The identities of participants were stored with each interview on a password-protected computer. Also, the researchers assured the participants that the obtained data would be used only for the study purposes, and all personal information would be omitted from any publications resulting from this study.

The researcher also offered much time for the participants as required to tell their stories and express their emotions. Talking about traumatic events can be therapeutic; however, it can also cause grief. Immediate support was offered to the participants, such as pausing the interview, switching off the audio-recorder, allowing the participants to cry and express their feelings, and providing supportive words in a compassionate way. A break was given for stressed participants, and they were offered the opportunity to reschedule the interview if they felt that they could not continue. The researcher stopped the interview immediately if any participant became too distressed, cried intensely, or reported being uncomfortable. In this study, most of the participants cried and reacted emotionally while recalling their experiences. To overcome the risk of emotional and psychological distress for the researchers, an expert psychological counselor was consulted when required.

### Pilot study

The first author (MMA) conducted one pilot interview with a female patient aged 64 years old, widow, retired, and diagnosed with multiple myeloma. After initial analysis of the audio recording, the author revised the interview guide and added questions on the difficulties after discovering the disease and on the resources that supported the participants.

### Rigor

Several steps were carried out to ensure the trustworthiness of findings [42]. First, participants were selected purposefully to ensure maximum variation, such as including both genders, various types of HMs, and both married and single participants. Also, the interviews were conducted by a trained researcher. Moreover, all researchers performed the data analysis and carefully examined the transcribed data to gain the essence of the studied phenomena. Finally, the interviews were transcribed for later analysis. The transcriptions were sent to each participant with the main results (themes and subthemes) to confirm that the researchers had captured the accurate meaning of their experiences and if there was any additional information that the participants wished to mention.

## Results

### The demographic of participants

The participants aged between 49 and 65 years. The majority (*n* = 6) were females. Two patients were diagnosed with leukemia and eight with lymphoma (of which six had non-Hodgkin and two had Hodgkin lymphomas) (Table 1). Time since receiving the diagnosis ranged between 12 months and 36 months.

**Table 1:** The demographics of participants

Name/ pseudonym	Gender	Age (year)	HMs subtype	Duration since diagnosis (months)	Length of the interview (minutes)
P1	Female	49	Lymphoma	14	60
P2	Female	56	Lymphoma	18	70
P3	Female	49	Leukemia	10	90
P4	Male	61	Lymphoma	42	70
P5	Female	50	Lymphoma	60	50
P6	Female	54	Lymphoma	84	70
P7	Female	58	Lymphoma	24	80
P8	Male	53	Leukemia	36	100
P9	Male	55	Lymphoma	30	70
P10	Male	65	Lymphoma	16	90

### Overview of the findings

The findings are described by two major themes. The first theme included twenty-one categories which were grouped into five subthemes and described pain, suffering, and distress (see Table 2). The second theme included nine categories grouped into three subthemes which described spiritual coping.

**Table 2:** The themes, subthemes, and categories

Themes	Sub-themes	Categories
**Theme 1: Pain and distress**	Sub-Theme 1: Dealing with constant, severe, and sudden pain	1. Inseparable part of life 2. Severe pain 3. Pain tolerance 4. Fluctuating pain 5. Sudden pain 6. Trying to live without drugs 7. Inadequate prescribing of opioids
	Sub-Theme 2: A debilitating body	1. Fatigue 2. Inability to perform daily living activities 3. Shortness of breath and insomnia 4. Lack of appetite 5. Lack of sexual relations
	Sub-Theme 3: Hopelessness	1. Pessimism and fear about the future 2. Fear of death 3. Losing interest in performing enjoyable activities 4. Losing family roles
	Sub-Theme 4: Social distress	1. Negative impact of the imminent death on family members. 2. Losing some friendships
	Theme 5: Spiritual distress	1. Lacking life purpose and meaning 2. God will help 3. Inability to pray
**Theme 2: Spiritual coping**	Sub-Theme 1: Wishing for a peaceful death	If one dies, it will be better for all Wish to die at home
	Sub-Theme 2: Accepting death	1. Death is one aspect of faith and destiny 2. Accept reaching the terminal stage of illness as a normal life cycle 3. Telling me to make my faith in Allah great
	Sub-Theme 3: Surrender to fate	1. Committed to praying 2. I must get closer to Allah 3. Submitting to fate 4. Affliction is as much as Allah loves us

### Theme 1: Pain, suffering, and distress

Most participants described several distressing symptoms that articulated their suffering as presented in the following subthemes.

#### Sub-theme 1: dealing with constant, severe, and sudden pain

In this theme, participants described suffering from pain that became an inseparable part of their life. Pain was the most common symptom reported by the participants. They described their suffering from pain with words such as “severe”, and “it is killing me”. This sense of severe pain was related to symptoms of HMs in the advanced stages of the illness. At the beginning of the illness, the participants received small doses of opioids. The doses were gradually increased according to the severity of pain. The participants’ tolerance of pain varied and increased gradually. Most participants reported that the severity of their pain fluctuated quickly. This is because they could not feel the gradual onset of mild pain due to the disease process and their increasing tolerance level; thus, they reported pain as sudden and intense. One participant said:

*“Oh God, please, can one month passes without pain! In the beginning, I took one tablet for analgesia, and then I started taking two pills together without relieving the pain. The pain in my back was increasing, despite taking Tramal, and I feel it is killing me.” (Participant 8)*.

The occurrence of sudden and severe pain could also be related to the delay in receiving analgesia, as some participants were trying to live without taking medications. The feeling of dependence on opioids was not easy for the participants and restricted their activities. One participant said:

*“I did not take my medication until 3 a.m. because I was tired of taking the treatment; I needed to sit down and relax without drugs. Suddenly, the pain increased more and more; then, I started screaming and crying. The pain became very intense and moved to my chest.” (Participant 7)*.

The severe pain could also be related to inadequate prescribing of opioids. Some participants said that when they visited the emergency department and requested opioids for their pain, some physicians refused to prescribe them due to fearing that the participants may become addicts or they were exaggerating the level of their pain. A participant said:

*“I used paracetamol for pain; the doctor gave it to me as an analgesic. He refused to give me opioids because he thought I was lying!” (Participant 1)*.

#### Sub-theme 2: a debilitating body

Most participants described a general sense of extreme fatigue which made them unable to perform the activities of daily living. They said that they could not sit up in a chair or go to the toilet. Most participants said that they felt tired and exhausted most of the day, every day. Two participants said:

*“Every day, I feel like I want to throw up, always tired, I want to stay in bed, and I always feel that the fatigue is killing me.” (Participant 9)*.

*“Everything changed a few months ago when I deteriorated; my movement became difficult and slow. I stopped being able to move, going outside the house, cooking, eating, cleaning, or using utensils. I stopped being able to do these things, which I did daily previously.” (Participant 3)*.

Some participants reported experiencing shortness of breath and insomnia. This is because they were terminally ill, and dyspnea and sleeplessness were some of the most common symptoms. The participants tried continuing with their lives despite the dyspnea. One participant said:

*“I cannot take a breath. I feel suffocated when I lay on my back, so I sleep on my side instead.” (Participant 8)*.

Furthermore, many participants reported losing appetite and did not feel like eating or drinking. The participants related feeling nauseated and losing their appetite due to pain, stress, or fatigue. A participant said:

*“At the beginning of my disease, my appetite for eating decreased, but now I cannot eat any food, even water. To the extent that once I smell the odor of food, I directly become nauseated and may even vomit.” (Participant 7)*.

Few participants reported a lack of sexual relationships with their partners because of their illness. They were very shy when they talked about this topic. They lowered their voices (although they were in a private location, i.e., their homes) and avoided eye contact with the researcher. They felt that their sexual dysfunction impaired their dignity and self-esteem, as this quote highlighted:

*“My wife told me things that are not acceptable in our culture for a wife to tell her husband! She told me that I am not a man, imagine! Because I have sexual weakness, and you know how much the chemotherapy and other treatments affect the human body.” (Participant 8)*.

#### Sub-theme 3: spiritual distress

The participants expressed a sense of pessimism and fear about the future due to the lack of cure. Participants said:

*“I was terrified, especially since I had finished all treatments. It occurred to me that I would die soon.” (Participant 2)*.

*“Every day I think that I will sleep and not wake up in the morning. My brain keeps thinking about what will happen to me.” (Participant 1)*.

The participants described a sense of fear of death, and that they felt powerless. Some participants cried when they talked about the future and said that they did not have any time left. The participants reported that they surrendered to death and lost interest in performing any enjoyable activities, like going to the garden or talking with friends. One participant said:

*“Sometimes, I resort to sleeping, and I keep thinking about my girls’ future. I have four daughters who need my support. Indeed, I know that my girls will be alone after my death. Because of this, I have no desire to go out or see anyone.” (Participant 9)*.

*“After the doctor informed me that there were no options to help me anymore, I did not sleep, and I cried a lot. I keep thinking more and more about my life before and after cancer. Really, I worry about dying suddenly at any moment.” (Participant 6)*.

Some participants expressed a lack of life purpose and meaning. Some participants wished they were dead. However, all participants believed that God would help them through this difficult time and would lead them to the best possible alternative. A participant said:

*“I do go to the mosque for praying, and sometimes, I think to stop everything: medications, praying, eating. But after that, I remember that this is considered suicide, and I believe this is wrong, and Allah will choose the best for me.” (Participant 10)*.

Other participants mentioned that they could not pray when they were very sick, which made them feel guilty. A participant said:

*“I am a Christian, I used to go to church every Sunday, but now I stopped going because I cannot drive, and at times I feel tired… I do not know if God will forgive me.” (Participant 5)*.

Other participants reported how they were unable to go to the mosque for praying and would sometimes delay praying due to fatigue and severe pain. One participant said:

*“When I hear the call for prayer in the mosque and I cannot go or pray due to severe pain, I feel guilty because in the past I could and now I cannot.” (Participant 7)*.

#### Sub-theme 4: social distress

Some participants were worried about the negative impact of their imminent death on their family members. Usually, family members are considered an important source of emotional and social support for terminally ill patients. However, family members can become a source of stress if they are mostly children who are cared for by the dying patient. A participant said:

*“I know that I am in the terminal stage, I keep thinking about what will happen to my children when I die, what will happen to these young children?” (Participant 8)*.

These feelings mainly led participants to lose their family roles. The participants said that they lost their ability to help themselves or support their families, which they said was very damaging to their self-esteem. The following quotations are examples:

*“In the last few days, I could not move my legs or body. When my sons carried me and put me in the shower to bathe me, I cried. I felt like a dead person, and I was sad because of the situation that I reached.” (Participant 4)*.

*“I cannot give my wife money… I feel that I lost my manhood.” (Participant 8).*

Other participants reported losing friendships and were worried about losing more of their social connections because of the disease. The participants said that their friends did not visit them often because they did not want to compromise their health or bother them; however, the participants felt that they had lost the support of their friends. A participant said:

*“My friends did not know that I had low immunity, and it started to go down a lot. When they found out about this, they stopped visiting me.” (Participant 10)*.

### Theme 2: spiritual coping

Most participants described different levels of spiritually based coping to the pain, suffering and distress presented in the first theme.

#### Sub-theme 1: accepting death

Some participants verbalized a wish to die peacefully. This could be related to uncontrolled severe pain, lack of support and motivation, and the feeling of being a burden to their family because of frequent hospital visits and admissions. One respondent said:

*“I know my condition and I say if one dies, it will be better for all…. My experience with severe pain makes me hope to die fast to comfort others because everyone will die at the end.” (Participant 10)*.

In addition to expressing a wish to die, most participants said that they would prefer to die at home rather than in hospitals. It is possible that the participants felt more comfortable with the home environment and more relaxed in a space free of devices, machines, and invasive procedures. Choosing home as a place for death in the terminal stage could be associated with acknowledging that no benefits from treatment remained and that they wished to see their family more often. In Arab culture, almost all family members are expected to be present with the patient to offer support and care [43]. In addition, most homes were adapted to be more comfortable and appropriate for the participants’ needs. One participant stated:

*“I do not like to sleep in the hospital area with others; for that, I like to stay at home and hope to die while I am at home as it is more comfortable for me.” (Participant 4)*.

Within the Islamic and Arabic cultures, receiving support is not limited to family members and healthcare providers but also includes neighbors, friends, and social systems [43]. Islamic and Arabic cultures motivate people to support each other in illness and wellness. Some participants reported that they talked with their family members and neighbors about dying, and that they supported and listened to their fears, as seen in the following examples:

*“My neighbors used to visit me in the hospital……they are afraid that I will die, and I am also afraid like them, but they were telling me to make my faith in Allah great, and they support me.” (Participant 2)*.

#### Sub-theme 3: surrender to fate

The patients’ faith in Allah led to accepting their illness. The patients expressed their surrender to fate and Allah’s plan. Muslims believe that only Allah can cure illness [44]. The following quotations reflect the participants’ submitting to fate:

*“One believes in Allah, and one does not have the power to change Allah’s choices, but one has to accept and cope with this illness. Allah decided that this is how it would happen to us.” (Participant 1)*.

*“Healing is in the hand of Allah, and this treatment is considered a way of healing. It may be healing or not. But Allah has everything in his hand.” (Participant 10)*.

*“At the beginning, I was a little upset, then I told myself that it was all from Allah and that it was all beautiful…and everything from Allah is definitely good.” (Participant 3)*.

Some participants had a higher level of spirituality than just their acceptance of the illness. They believed that their illness and EOL care were the best things that could happen because that was what Allah chose for them. In Islam, the person who suffers receives more compensation from Allah in the afterlife. Muslims believe that the greater an affliction is, the greater is Allah’s love [44]. Accordingly, as shown in the next quotes, participants perceived their illness as good and the best thing that could happen for them:

*“Allah knows I am satisfied with the disease, and I am satisfied because this is the best thing that could happen to me.” (Participant 8)*.

*“This disease is from Allah, and this is good because Allah always chooses the good for the person, and surely our Lord sees that this is better for me.” (Participant 1)*.

*“Praise be to Allah. This disease, Allah choose it for me, and I am happy with it.” (Participant 3)*.

*“This disease is all from Allah, and Allah tests each person on the extent of Allah’s love for him.” (Participant 2)*.

#### Sub-theme 2: looking forward to the afterlife

Most participants believed that they had reached the terminal stage, and that death was imminent. In Islam, believing in a life after death is an important aspect of faith. Most participants believed in life after death and in Paradise. They performed Islamic rituals that kept them close to Allah and helped them face their illness. This gave them a sense of reassurance and relaxation:

*“I am committed to praying, reading the Qur’an, and other things to be close to Allah.” (The Participant 10)*.

*“Sometimes I go to pray in the mosque…… frankly I cannot read Qur’an frequently, because I have severe fatigue and exhaustion that broke my back. I adhere to praying…I must get closer to Allah.” (Participant 8).*

*“The most important thing is that a person remains as close to Allah as he can, because one does not know when his life would end.” (Participant 2)*.

“I always ask Allah’s forgiveness, and I always have my Masbaha*, and I keep praying to Allah in these virtuous days that I come back home well.” (Participant 6).

* Masbaha is a tool used to engage in praying.

## Discussion

Understanding the experience of patients with HMs in the terminal stages is an understudied phenomenon that requires further research, particularly in the developing world, such as in Arabic countries. This study aimed to explore the experience of Jordanian adults with HMs and provide contributions to existing knowledge about distressing symptoms that continue throughout the terminal stages of their illness. All patients in this study have suffered from severe, constant, and sudden pain. This finding is congruent with previous studies of patients in the terminal stages of HMs [37, 45]. Having severe uncontrolled pain in the terminal stage of cancer could be related to inadequate prescription of opioids by physicians [46]. In Jordan, inadequate pain management is a problem [47, 48]. There has been a lack of studies of patients with HMs; however, a recent study of patients with solid cancers reported that more than 80% of cancer patients experienced poor pain management as a result of inadequate prescription of opioids and non-opioid drugs [47]. Cultural or legal issues often interfered with prescribing opioids for cancer patients in Jordan [48].

Our findings highlighted other significant distressing symptoms, such as fatigue, anorexia, insomnia, and sexual dysfunction. The literature reports that HMs patients suffer from different symptoms with varying severity according to the nature and stage of their particular HM, and side effects of the therapeutic regimens [6, 49, 50]. These findings were not surprising, as this group of patients is in the terminal stages of illness and receive multiple medications. Symptoms become more burdensome and stressful over time, as previously reported in the literature [45, 51, 52]. Furthermore, our participants who were diagnosed with cancer a long time ago (e.g., Participant 7 and 8) were suffering from severe uncontrolled pain in addition to worsening of other symptoms and social distress. This finding is supported by previous studies [8, 52–54]. Those two participants were readmitted to hospital three weeks after the interview and died there. Healthcare providers should be aware of the impact of poor pain management on patients in aggregating the distressing symptoms which continues until death.

Besides physical discomfort, many of the participants in our study highlighted psychological and emotional distress including fear of the future and death; fear of losing their family roles; and lack of enjoyment. Experiencing such feelings is mostly related to poor prognosis, treatment failure, or physical symptom burden [55], which increased fear of the future, especially with no further treatment options available. In addition, the participants reported that they were unable to perform activities of daily living due to fatigue and general weakness. In Arabic culture, the parent’s role is presented as an image of hero in the family [43]. Parents must protect their family in wellness and in illness, and if anyone becomes ill, all family members should stay around the ill member. In Arabic culture, this is the goal of parenting. When cancer patients become severely ill, they become unable to care for their families, which adds to the negative impact of the disease. The participants’ fear of losing their family roles is related to the Arabic culture which depicts fulfilling these roles as loyalty to their family [43]. Furthermore, as HMs progress with limited curative options, patients can become more emotionally distressed and experience feelings of helplessness, frustration, and fear of death, which could lead them to lose hope of cure [56, 57]. A recent qualitative study conducted by Boucher et al., (2018) reported that patients with advanced stages of leukemia lost hope for a cure and accepted the idea of scarce curative options in addition to other physical and psychosocial distresses [57]. Our findings emphasize the feeling of powerlessness and loss of hope among terminally ill HMs patients, which influenced their roles in life, at home, and in society.

Globally, it is known that the availability of a supportive social system around cancer patients has a significant positive impact on their overall quality of life. For patients with HMs, the role of the family, friends, and other supportive systems is invaluable and considered an essential lifeline that should be integrated with care plans [56]. Some participants in our study reported that they lost friendship due to their disease progression and the imminence of their death, which also increased their negative psychological impacts in addition to their physical distress. A study found that the unmet psychosocial needs of HMs patients were dominant unmet needs [58]. Loss of social support can cause loss of hope and loss of the ability to cope with illness [36, 57]. It is therefore important to maintain a strong social support system around HMs patients to minimize their distress and give them hope.

As human beings are composed of physical, psychological, and spiritual elements, any physical or psychological distress experienced by HMs patients can also affect their spiritual well-being [34, 35]. In our study, participants highlighted how the accumulation of negative physical, psychological, and social distress led to spiritual distress. In Arabic countries, the majority of the population are Muslims, and their faith in Allah is a fundamental aspect of their lives [44]. However, when patients face great, uncontrollable, distressing symptoms with a poor prognosis, it can challenge their hope for a cure and ultimately impact their sense of purpose in life. While faith can provide comfort and support, spiritual distress can still arise in the face of such profound suffering [35].

A recent systematic review reported that spiritual wellbeing is influenced positively by feelings of happiness, life satisfaction, and proper management of physical symptoms [59]. Patients with HMs, like other cancers, have spiritual needs and distresses to which the contribution of physical and psychological factors is enormous. This explains why the participants in our study had several spiritual stresses besides their other stressors. This is consistent with the literature regarding spiritual distress among patients with HMs; this could support the need to address all patient needs from simple symptoms and to hidden feelings; to maintaining hope and meaning in life [57].

Another significant finding of our study was that most participants expressed surrender to fate and Allah’s plan. Muslim people have faith in Allah as they surrender to fate and accept all of Allah’s arrangements. Some Muslims perceive their illness and EOL stage as the best things that could occur for them, because Allah chose this. In the Islamic readings of the Qur’an and Sunnah, the person who experiences more suffering and distress will receive more compensation from Allah in the hereafter [44, 60, 61]. Muslims believe that affliction is a sign of Allah’s love. These findings are congruent with several studies of Muslim cancer patients [34, 62, 63]. A recent integrative review was conducted to describe the psychosocial and spiritual outcomes and perspectives among Muslim patients with cancer who received treatment [62]. The findings of this review revealed that cancer patients rely on their faith in Allah to maintain spiritual well-being, and that they also depend on their belief in Allah as the source of power which gives them a sense of inner strength when reading the Qur’an [64]. Muslims usually look for treatment when they become ill, but they accept death as the end process of life. Thus, integrating spiritual support within the care for terminally ill patients would reduce their emotional distress and improve their quality of life [44].

Once the participants reached a stage in which they were waiting for the last moments of their life, they wished for a good death, incorporating their distress with faith in Allah’s fate for them. The term good death can be conceptualized differently across cultures and diseases, but in general a good death means being free of pain; not being a burden to others; good family and social support; psychological comfort; spiritual peacefulness; unchanged religious belief; and valuing the meaning of life [65–67]. These findings are consistent with our findings of participants’ wishes to die to alleviate pain and suffering, and to avoid burdening others. When cancer patients reach the terminal stage, they often feel like a burden to those around them. This can result in strong negative emotions and even suffering, as they believe that they are a burden to their caregivers [68]. It is crucial to understand that these feelings may arise due to various factors, such as psychological distress, social distress, illness trajectories, gender issues, and sociocultural background, and not just the patient’s own assumptions.

The meaning of a good death was associated with participants’ wishes to die at home rather than in the hospital, as they felt more comfortable in the home environment and more relaxed in a space free of devices, machines, and invasive procedures. This is congruent with previous studies, which showed that a significant component of a good death was the wish to die at home. This is due to the important role of the home in offering a comfortable environment for loved ones to stay nearby and to spend more time with them [65, 66, 69].

There is evidence of a connection between religious beliefs and end-of-life care. Patients who have a strong religious faith or spiritual values are more likely to engage in advanced care planning activities and make supportive end-of-life care decisions [70, 71]. From the Islamic perspective, these beliefs can help patients accept the inevitability of death and provide emotional and spiritual comfort during the dying process [62, 63]. However, although most of the patients in the current study were Muslims and advanced care planning is a part of their religious rituals, no one had discussed advanced care planning issues. A multicenter study conducted in Jordan among dying cancer patients reported that rigorous treatment was provided in hospitals in the last few days of their lives, emphasizing the importance of discussing advance care planning with terminally ill cancer patients [72].

### Implications for practice, policy, education, and future research

Our study offers foundations for guiding further research to explore the lived experiences of patients with HMs in the terminal stage of illness in Jordan. One of the most significant findings of this study is the inadequate pain management and ongoing patient suffering despite reaching the advanced stages of HMs. Proper pain management protocols should be taught to healthcare providers who deal with HMs patients, emphasizing the provision of individualized care. Our findings also emphasize the importance of providing appropriate symptom management for HMs patients in Jordan. In hematology settings, nurses and hematologists need to assess the physical, psychosocial, and spiritual needs of patients and aim to achieve them based on a patient-centered approach.

Offering education programs for healthcare providers to focus on the bio-psychosocial dimensions of care and the patient’s experience of the HMs would improve care. Thus, academics and clinicians must act collaboratively to develop these educational programs and overcome the gap between academic researchers and healthcare providers who serve in the clinical area regarding the provision of adequate pain and symptoms management. In terms of pain management, exploring the perspectives of healthcare providers regarding opioid prescriptions and restricting increased opioid doses is important Further longitudinal, prospective studies are required to recognize the bio-psycho-social suffering, quality of life changes, and supportive care needs among patients with HMs during the terminal stage of their illness. Also, a further emphasis on this aspect of care should be extended to include a spiritual guide in hospitals to provide spiritual support for patients.

### Recommendations

Few studies have been conducted in Jordan on HMs patient experiences of pain and distress at the terminal stage. Thus, to improve the experiences of patients in Jordan, further research is recommended to assess their needs; provide suggestions on proper symptom management; and when to make referrals to specialized pain management or palliative care services. More qualitative studies must be conducted among Jordanian patients with HMs and healthcare providers to understand how to manage patients from their perspective. Policy makers should establish evidence-based protocols for providing HMs patient care and discussing advanced care planning with terminally ill patients. They should also consider establishing a national collaborative program aiming to enroll qualified healthcare providers to address the bio-psycho-social and spiritual needs of patients and manage their distressing symptoms appropriately. Further follow-up and longitudinal studies are required to evaluate the relationship between the patient distress, patient outcomes, and quality of life.

### Strengths and limitations

This study explores pain and distress among patients with HMs from different cancer centers. It can be used to support evidence-based practice to address patient needs and improve and coordinate care services between healthcare providers and patients. However, this study has limitations. The study was conducted among ten participants who had shared experiences, while other patients in different study settings may have expressed different views. Also, the study was conducted in two different settings (patients’ homes and hospital), which could be associated with potential bias. The sample size was small, although this is a general limitation of qualitative research. Only one patient of Christian faith was included, which made it difficult for the researchers to understand the spiritual distress and coping across different religions, as the majority of the participants were Muslim. Including patients from different religions in further studies would be beneficial. Also, many patients were approached and couldn’t participate in the current study due to deteriorations in their health status or severe psychological distress, as they were in the terminal stage of illness. In addition, the participants had different forms of HMs; this highlighted the importance of exploring experiences within specific types of HMs. This was a short-term study and conducting long-term and repeated interviews among patients would be a valuable future study design.

## Conclusions

This study used a phenomenological approach to provide a comprehensive and in-depth understanding of the experience of pain and distress among patients with HMs. The participants reported suffering from severe pain and several physical, psychosocial, and spiritual symptoms. During their journeys with HMs, negative biopsychosocial suffering was the dominant experience among participants. Addressing the terminal stage of illness and providing care in a more organized manner is important for healthcare providers to address patient needs from commencing care until they reach a peaceful death. Considering spiritual needs and religious care is important for HMs patients. This study provides essential data for further expanding end-of-life and palliative care services in Jordan and other Middle Eastern countries.

### Electronic supplementary material

Below is the link to the electronic supplementary material.

Supplementary Material 1

## Data Availability

All data generated or analyzed during this study are included in this published article.
