# Supplementary material for: The experience of patients with hematological malignancy in their terminal stage: a phenomenological study from Jordan’s perspective
Source: BMC Palliat Care. 2024 Feb 9;23:36. doi: 10.1186/s12904-024-01373-y (PMC10854087; doi:10.1186/s12904-024-01373-y)
Supplement: Supplementary file 1 — Supplementary Material 1 [file 12904_2024_1373_MOESM1_ESM.docx]

**Supplementary Table: Interview guide for participants**

| **Sub-questions and interview questions** | |
| --- | --- |
| Can you describe your experience after the disease was discovered? | How difficult was it for you to be faced with the disease? Can you describe your feelings during that situation? What stressors did you face? |
| Where are there needs either met or not during this period? | Can you tell me how you dealt with the disease after discovering it? What did help you? What were the sources of strength? |
| Which situations were difficult for you after the diagnosis was discovered? |  |
